# Comparison of four culture protocols for differentiating bovine peripheral blood mononuclear cells into macrophages

**DOI:** 10.3389/fvets.2026.1851348

**Published:** 2026-06-23

**Authors:** Sang Young Seo, Han Gyu Lee, Young-Hun Jung, Bock-Gie Jung, Yoon Jung Do, Are-Sun You, Eun-Yeong Bok

**Affiliations:** 1 Division of Animal Diseases & Health, National Institute of Animal Science, Rural Development Administration, Wanju-gun, Jeonbuk-do, Republic of Korea; 2 Department of Veterinary Microbiology, College of Veterinary Medicine, Chonnam National University, Gwangju, Republic of Korea

**Keywords:** bovine macrophages, macrophage differentiation, *Mycobacterium avium* subsp. paratuberculosis (MAP), peripheral blood mononuclear cells, phenotype

## Abstract

**Introduction:**

Macrophages are essential innate immune cells involved in host defense, tissue homeostasis, and inflammation regulation. *In vitro* differentiation protocols for macrophages derived from peripheral blood mononuclear cells (PBMCs) are well established in human and murine models. However, in bovine species, standardized protocols for macrophage differentiation from PBMCs are underdeveloped, limiting their application in immunological and infectious disease studies.

**Methods:**

Four *in vitro* culture protocols for differentiating macrophages from bovine PBMCs were compared, with the aim of comparing their morphological, phenotypic, and phagocytic characteristics. PBMCs were isolated from clinically healthy cattle and cultured under four protocols: (1) macrophage colony-stimulating factor-supplemented RPMI-1640, (2) nutrient-enriched RPMI-1640 without exogenous cytokines, (3) commercial M1-inducing medium, and (4) commercial M2-inducing medium.

**Results:**

Differentiated macrophages were evaluated using phase-contrast microscopy, quantitative reverse transcription polymerase chain reaction, and flow cytometry for surface marker expression, and phagocytic assays using heat-killed *Mycobacterium avium* subsp. *paratuberculosis* (MAP). Under protocol 2, macrophages exhibited more pronounced morphological differentiation and higher expression of CD14, CD11b, and MHC II compared to other conditions, suggesting a more differentiated phenotype. This phenotype was associated with increased phagocytic activity, as evidenced by the highest phagocytic activity against MAP.

**Discussion:**

Overall, these findings suggest that nutrient-enriched RPMI-1640 medium without cytokine supplementation (protocol 2), a commonly used culture condition, supports macrophage differentiation with favorable morphological and phagocytic characteristics under our experimental conditions. This culture approach provides a valuable *in vitro* model for studying bovine immunology and macrophage–pathogen interactions.

## 1 Introduction

Macrophages are multifunctional immune cells (1, 2) that possess the plasticity to polarize into two major phenotypes: M1 macrophages, which primarily promote inflammation and defend against pathogens, and M2 macrophages, which are involved in suppressing inflammation, tissue regeneration, and immune regulation (3–6). The transition of circulating monocytes into these diverse macrophages is regulated by complex microenvironmental signals, including growth factors, cytokines, and nutrient availability (7, 8). However, recent studies in bovine models have demonstrated that macrophage polarization is not fixed. Instead, bovine macrophages exhibit substantial phenotypic plasticity and functional heterogeneity depending on environmental conditions, pathogen exposure, metabolic regulation, and the inherent diversity of circulating monocyte subsets (9–12). These variations are directly associated with distinct functional responses, highlighting the complexity of macrophage differentiation in bovine systems (13, 14). Similar observations have also been reported in studies describing heterogeneous macrophage differentiation and activation states under distinct culture conditions (15, 16). Therefore, establishing suitable *in vitro* differentiation protocols that accurately reflect this phenotypic plasticity is crucial for advancing our understanding of the bovine immune system and formulating disease control strategies.

Cattle are highly susceptible to intracellular pathogens, including those causing tuberculosis and paratuberculosis. In these infections, macrophages serve not only as the primary host niche for bacterial replication but also as the first line of defense (12). In particular, *Mycobacterium avium* subsp. *paratuberculosis* (MAP), the causative agent of paratuberculosis, primarily targets macrophages to persist and evade immune surveillance (17, 18). Thus, establishing a functional *in vitro* macrophage model is essential for investigating host immune responses against these intracellular threats.

To study these macrophage-mediated immune responses, researchers require reliable cell culture systems. Previous studies have focused on differentiating macrophages from bovine bone marrow cells using macrophage colony-stimulating factor (M-CSF), reporting high differentiation efficiency and cell viability (19). However, bone marrow collection is invasive and impractical for repeated sampling, limiting its use in longitudinal studies (20, 21). In contrast, PBMCs can be readily obtained from peripheral blood in large quantities, making them suitable for repeated and large-scale experiments (10, 14, 22). For culturing these cells, RPMI-1640 is widely used for the culture of PBMCs and monocyte-derived macrophages because it was originally formulated to support the growth and maintenance of hematopoietic and suspension cells under *in vitro* conditions (10, 13, 14, 22). Indeed, several studies have successfully utilized bovine peripheral blood monocyte-derived macrophages (PBMC-MDMs) to investigate various immunological mechanisms, including host responses to bacterial infections and metabolic immunomodulation (9, 11, 15, 16). However, these studies have employed a wide variety of culture conditions. Given the substantial phenotypic plasticity of bovine macrophages, differing culture environments can lead to highly heterogeneous differentiation and activation states. While extensive research on human and murine models has established well-characterized *in vitro* differentiation protocols for macrophages derived from PBMCs (23, 24), these methods may not directly translate to cattle. This discrepancy is largely due to species-specific differences in cytokine receptor compatibility and signaling pathways (10, 22). Therefore, further refinement of species-specific differentiation protocols for bovine macrophages is critically needed, particularly to better reflect the heterogeneous and context-dependent nature of macrophage differentiation in cattle.

In this study, four culture conditions to induce macrophage differentiation from bovine PBMCs were compared with the aim of establishing the optimal protocol based on morphological, phenotypic, and functional characteristics. The culture conditions were bovine M-CSF-supplemented medium, nutrient-enriched RPMI-1640, and two commercially available media for human M1 and M2 polarization. The findings lay a foundation for in-depth studies into the immune mechanisms underlying key bovine diseases, such as paratuberculosis, thus contributing to the development of improved diagnostic, preventive, and therapeutic strategies in veterinary medicine.

## 2 Materials and methods

### 2.1 Experimental animals

All procedures involving animals were approved by the Animal Ethics Committee of the National Institute of Animal Science (NIAS), Republic of Korea (approval no. NIAS 2022–0559). Blood samples were collected from 22, clinically healthy, Hanwoo cattle that were randomly selected from a farm located in Wanju-gun, Republic of Korea. The animals had a mean age of 76 months (range: 33–122 months) and included both males (n = 5) and females (n = 17). All selected animals tested negative for foot-and-mouth disease, brucellosis, and bovine tuberculosis before sample collection, and were seronegative for paratuberculosis based on a commercially available ELISA kit (Paratuberculosis Verification Ab Test, IDEXX Laboratories, Inc., Westbrook, ME, USA). For peripheral blood mononuclear cell (PBMC) isolation, 30 mL of blood was drawn once per animal from the jugular vein using a sterile, disposable syringe equipped with an 18-gauge needle (Korea Vaccine Co., Ltd., Republic of Korea) and transferred into heparinized blood collection tubes (BD Vacutainer, containing lithium heparin). PBMCs isolated from individual animals were considered independent biological replicates. For each experiment, PBMCs from the same donor were subjected to all four culture conditions (P1–P4) in parallel. Experiments were repeated using cells derived from at least three to five different animals. Only animals that tested negative for all screened pathogens and showed no clinical signs of infection were included in this study.

### 2.2 Isolation of PBMCs

PBMCs were purified using LeucoSep tubes (Greiner Bio-One, Kremsmünster, Austria) according to the manufacturer’s instructions. Briefly, 15 mL of Ficoll-Paque PLUS (Cytiva, Chicago, IL, USA; density, 1.077 g/mL) was preloaded into a 50-mL LeucoSep tube and centrifuged at 1,000 × *g* for 30 s using an Allegra X-15R centrifuge (Beckman Coulter, Brea, CA, USA). For density gradient separation, blood from each animal was diluted 1:1 (*v/v*) with Dulbecco’s phosphate-buffered saline (DPBS; Thermo Fisher Scientific, Waltham, MA, USA), and 30 mL of the diluted blood was carefully layered onto the LeucoSep tube. The tubes were centrifuged at 800 × *g* for 15 min at 25 °C without braking to allow efficient separation of mononuclear cells while minimizing cell activation and mechanical stress. The PBMC layer was collected, and the cells were washed twice with DPBS (400 × *g*, 10 min, 25 °C) to remove platelets and residual Ficoll. Subsequently, the cells were resuspended in Roswell Park Memorial Institute (RPMI)-1640 medium containing L-glutamine and HEPES with manufacturer’s standard glucose concentration (Gibco, Grand Island, NY, USA; Cat#22400–089), supplemented with 10% (*v/v*) heat-inactivated fetal bovine serum (FBS; Gibco; Cat#10082–147) and 1% penicillin–streptomycin (PS; Gibco; Cat#15140–122). Live cells were counted using trypan blue exclusion and a Countess 3 automated cell counter (Invitrogen, Waltham, MA, USA); cell viability typically exceeded 90%.

### 2.3 Macrophage culture and differentiation

PBMCs were adjusted to a density of 1 × 10^6^ cells/mL in complete medium and seeded into 24-well plates (Corning, NY, USA). Four different complete culture conditions were used to induce monocyte-to-macrophage differentiation: (P1) M-CSF-supplemented medium: RPMI-1640 containing L-glutamine and HEPES, 10% FBS (Gibco), 1% PS (Gibco), and 50 ng/mL bovine M-CSF; (P2) nutrient-enriched RPMI-1640: RPMI-1640 containing L-glutamine and HEPES, 1% MEM non-essential amino acid solution (Thermo Fisher Scientific Inc., Waltham, USA; Cat#11140–050), 2% MEM essential amino acid solution (Thermo Fisher Scientific; Cat#11130–051), 2 mM L-glutamine (Gibco; Cat#11360–070), 1% sodium pyruvate (Thermo Fisher Scientific), 50 μM 2-mercaptoethanol (Sigma-Aldrich, St. Louis, MO, USA), 5% FBS (Gibco), and 1% PS (Gibco); and (P3) M1-Macrophage Generation Medium XF (PromoCell GmbH, Heidelberg, Germany), a commercial medium containing recombinant human GM-CSF and IFN-*γ* according to the manufacturer’s documentation; and (P4) M2-Macrophage Generation Medium XF (PromoCell GmbH), a commercial medium containing recombinant human M-CSF and IL-4 designed for human monocyte polarization.

24-well plates (Corning) were incubated at 37 °C in a humidified incubator with 5% CO₂. After an initial 2 h adherence period, non-adherent cells were gently removed by washing twice with warm DPBS, and adherent cells were cultured in fresh medium corresponding to each condition. To support the high metabolic demand and prevent the potential accumulation of toxic ammonia resulting from glutamine degradation, the culture medium was replaced every 2–3 days. Cells were maintained for a total of 10 days to allow full maturation of macrophage morphology and surface marker expression. Adherent macrophages were detached using 0.25% trypsin–EDTA prior to downstream analyses.

### 2.4 Macrophage morphology quantification

PBMC-MDMs were washed with DPBS, and their morphology was assessed using an Olympus IX73 inverted microscope (Olympus Corporation, Tokyo, Japan). High-resolution images were captured on day 10 of cell culture using a × 100 objective in phase-contrast mode, and analyzed using ImageJ software (version 1.53e; National Institutes of Health, USA). For each condition and experiment, at least five randomly selected fields per well from three wells per condition were quantified, and the analysis was conducted across three to five independent biological experiments. Cells were classified into three morphological categories based on previously described criteria (25): rounded/less-spread cells, “fried egg”-like cells with a large cytoplasmic area surrounding the nucleus, and elongated/spindle-like cells. Morphological categories were interpreted in conjunction with phenotypic marker expression and not as an isolated proxy for maturation status. Data were collected from three to five independent biological experiments using cells cultured in the four different media. For qualitative morphological confirmation, cells were additionally subjected to Giemsa staining. Briefly, cells were fixed with 100% methanol for 5 min and stained with 10% Giemsa solution (Biognost, Croatia) for 20 min. After rinsing with distilled water, stained cells were examined under the same microscope at ×400 magnification to assess cellular morphology and nuclear features. This staining was used for descriptive observation only and was not included in quantitative morphological analysis.

### 2.5 RNA purification and reverse transcription–quantitative PCR (RT-qPCR)

Total RNA was extracted from PBMC-MDMs using the RNeasy Mini Kit (Qiagen, Hilden, Germany) according to the manufacturer’s protocol. Cells were lysed directly in culture wells with Buffer RLT supplement with 10% *β*-mercaptoethanol, and lysates were collected by scraping. RNA concentration and purity were measured using an Epoch Microplate Spectrophotometer (BioTek Instruments, Winooski, VT, USA), with RNA quality assessed using A_260_/A_280_ ratio (acceptance criteria: 1.8–2.1). Samples were stored at −80 °C until further use.

The complementary DNA was synthesized from 500 ng of total RNA using the HisenScript RH (−) RT PreMix Kit (Invitrogen, Carlsbad, CA, USA), following the manufacturer’s instructions (60 °C for 1 h). Specific primers for genes of interest were designed using Primer3 software (version 0.4.0), and primer sequences are listed in Table 1. Real-time qPCR was conducted using an ABI 7500 Real-Time PCR System (Applied Biosystem, CA, USA) with 1 × SYBR green PCR master Mix (Thermo Fisher Scientific) and 10 μM of each primer pair in a 20-μL reaction volume.

**Table 1:** List of sequences of primers used for quantitative reverse transcription polymerase chain reaction.

Gene	Primer Sequence (5′-3′)	Accession number	bp
*ACTB*	F CTCTTCCAGCCTTCCTTCCT R GGGCAGTGATCTCTTTCTGC	NM_173979.3	178
*CD86*	F TTTTCAGGTGCTGCTTCCTT R CAAGGTCCAACTGTCCTGGT	NM_001038017.2	228
*CD14*	F GCAGCCTGGAACAGTTTCTC R TCCTCAAGCGTCAGTTCCTT	NM_174008.1	178
*CD163*	F CGAGTCCCATCTTTCACTCTG R AGTGAGAGTTGCAGAGAGGTCC	NM_001163413.1	184
*CD11b*	F ACCCAGTGGTAAAAGGCCAG R GACGTGTAGAGACGCTGAGG	NM_001039957.1	200
*MHC II*	F CAGATCAAGGTTCGGTGGTT R TCACGAGGATCTGGAAGGTC	NM_001034668.3	103

ACTB, β-actin; CD, cluster of differentiation; MHC II, major histocompatibility complex class II.

The thermal cycling conditions were: 95 °C for 10 min, followed by 40 cycles of 95 °C for 15 s and 60 °C for 1 min. Primer specificity was confirmed by melt curve analysis, and no-template controls were included in each run to exclude contamination. Relative gene expression was calculated using the ΔΔCt method (26) and normalized to the housekeeping gene, *β-actin* (*ACTB*). *ACTB* was selected as reference gene based on its reported stable expression in bovine immune cells (27), and its consistent Ct values across the experimental conditions in this study. All experiments were conducted in triplicate.

### 2.6 Flow cytometric analysis

On day 10 of differentiation, undifferentiated monocytes and macrophages differentiated under the four distinct conditions were gently harvested by washing with cold DPBS and detached using 0.25% trypsin–EDTA. Cells were then transferred into polystyrene flow cytometry tubes at a concentration of 1 × 10^6^ cells per tube. Subsequently, cells were stained with primary antibodies or corresponding isotype controls (Table 2) in the dark at 4 °C for 30 min. After two washes with FACS buffer (Ca^2+^/Mg^2+^-free PBS supplemented with 2% FBS and 2 mM EDTA), cells were incubated with appropriate fluorochrome-conjugated secondary antibodies under the same conditions for an additional 30 min. Labeled cells were fixed with 1% paraformaldehyde. For data acquisition and analysis, cellular debris was excluded based on forward scatter (FSC) and side scatter (SSC) characteristics, and singlet cells were gated using FSC-A versus FSC-H parameters. No viability dye was used; therefore, dead cells were excluded based on scatter properties. Compensation was conducted using single-stained controls. At least 10,000 events were analyzed using the Bigfoot Spectral Cell Sorter (ThermoFisher Scientific). Flow cytometry data were processed and analyzed using FlowJo software version 10.10 (FlowJo LCC, Ashland, USA). The expression levels of CD14^+^, CD86^+^, and CD163^+^ were quantified based on fluorescence intensity measurements under each experimental condition. Fluorescence thresholds were set based on isotype control signals, and results are presented as geometric mean fluorescence intensity (gMFI) values rather than percentage of positive cells. Fluorescence intensity values were calculated as geometric mean fluorescence intensity (gMFI) after gating on singlet cell populations.

**Table 2:** List of antibodies used for flow cytometry.

Antigen	Target species	Clone	Isotype	Dilution	Source
CD14, FITC	Mouse anti-bovine	CC-G33	IgG1	1:250	Bio-Rad
CD11b, Alexa fluor® 647	Mouse anti-bovine	CC126	IgG2b	1:250	Bio-Rad
CD163, unconjugated	Mouse anti-bovine	EDHu-1	IgG1	1:250	Novus
MHC II, unconjugated	Mouse anti-bovine	CC108	IgG1	1:250	Bio-Rad
CD86, FTIC	Mouse anti-bovine	IL-A190	IgG1	1:250	Bio-Rad
ABflo 594-conjugated Goat anti-Mouse IgG	Mouse IgG1	polyclonal	IgG1	1:500	ABclonal

FITC, fluorescein isothiocyanate; ABflo 594, Alexa Fluor 594.

### 2.7 Phagocytic activity assay

Differentiated PBMC-MDMs cultured in 24-well plates were infected with heat-killed *Mycobacterium avium* subsp. *paratuberculosis* K-10 at a multiplicity of infection of 10:1. The cells were then incubated for 2 h at 37 °C in an atmosphere enriched with 5% CO₂. After incubation, cells were washed twice with pre-warmed DPBS and subsequently treated with culture medium containing 10 μg/mL gentamicin (ThermoFisher Scientific) for 2 h to eliminate extracellular bacteria. Cells were then fixed with 100% methanol for 5 min and air-dried at 25 °C. Ziehl–Neelsen staining was conducted using the TB-Stain Quick Kit (Biognost, Croatia), following the manufacturer’s instructions. Briefly, fixed cells were stained with TB carbol fuchsin for 5 min with intermittent heating, decolorized with TB decolorizer for 30 s, and counterstained with TB Armand reagent for 3 min. For quantitative analysis, images were acquired at 400 × magnification from four distinct fields per well using an Olympus BC53 microscope (Olympus, Japan), and the analysis was conducted across three independent biological experiments. The number of infected and uninfected cells was quantified using Image J software. Cells containing one or more intracellular acid-fast bacilli were defined as phagocytic cells, whereas cells without visible bacilli were considered non-phagocytic. Bacteria attached to the cell surface without clear internalization were excluded from analysis. Phagocytic percentages were calculated and expressed as mean ± standard deviation (SD) from three independent experiments (Equation 1):


$$\begin{array}{l} \text{Phagocytic percentage}=(\text{Number of phagocytic cells}/\\ \;\;\;\;\;\;\;\;\;\;\;\;\;\;\;\;\;\;\;\;\;\;\;\;\;\;\;\;\;\;\;\;\;\;\;\;\;\;\;\;\;\;\;\;\;\;\;\;\;\;\;\;\;\;\;\text{Total number of cells})\times 100 \end{array}$$ (1)

### 2.8 Statistical analysis

Statistical analyses were conducted using Prism software (version 9; GraphPad Software, San Diego, CA, USA). All data are presented as mean ± standard deviation (SD). Before conducting a one-way analysis of variance (ANOVA), normality was assessed using the skewness and kurtosis values in SPSS software (version 27.0; IBM Corp., USA). Differences among the four independent groups were evaluated using a one-way ANOVA, followed by Tukey’s honestly significant difference (HSD) post-hoc test. Biological replicates (independent animals) were used for all statistical analyses. All experiments were conducted in triplicate or more, and *p* < 0.05 was considered statistically significant.

## 3 Results

### 3.1 Macrophage morphology

**Figure 1:**
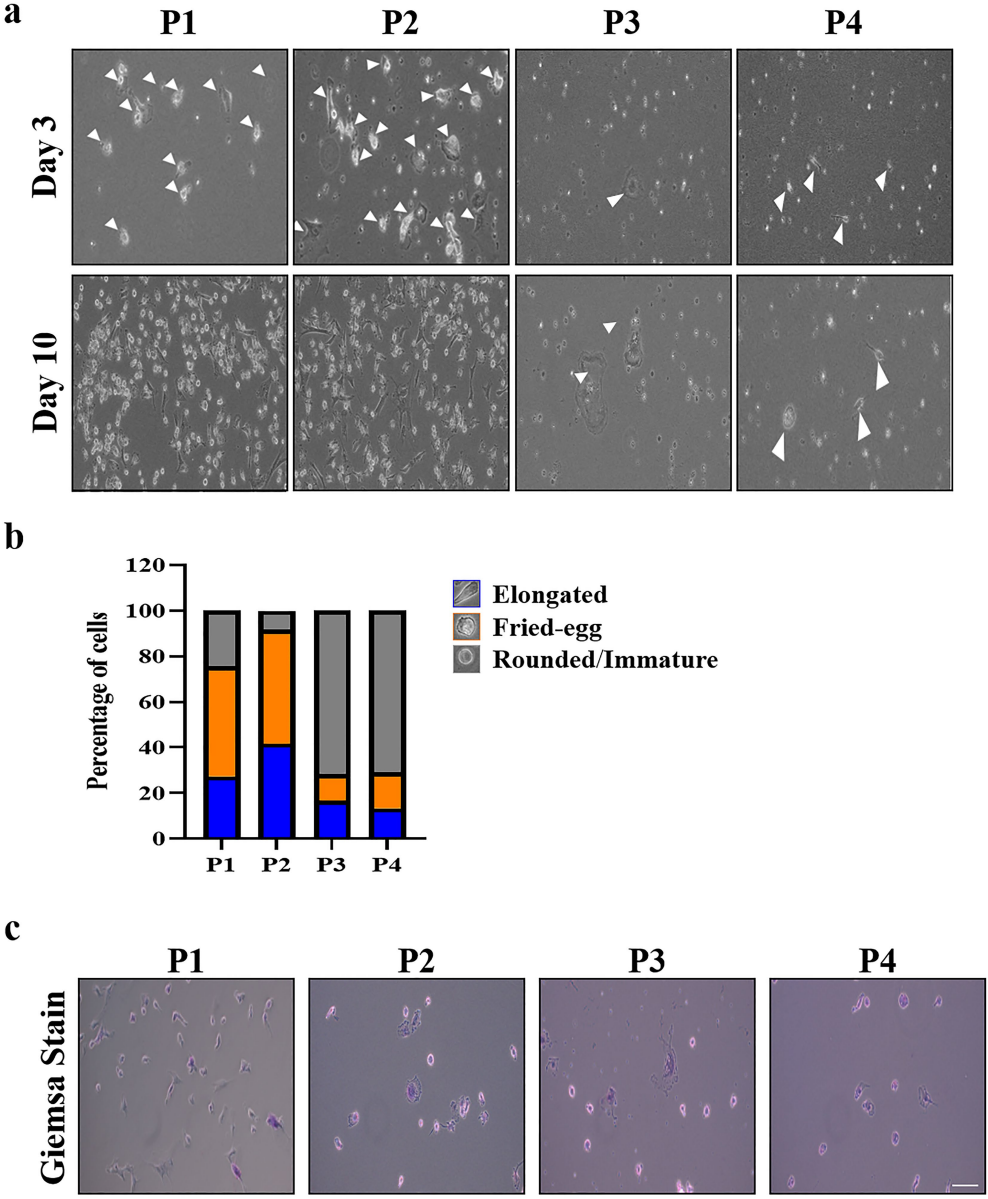
Analysis of cell morphology and staining variations under different protocols. **(a)** Time-lapse phase-contrast images of adherent cells differentiated under four protocols (P1, M-CSF-based medium; P2, nutrient-enriched cytokine-free medium; P3, commercial human M1-polarizing medium; and P4, commercial human M2-polarizing medium) on days 3 and 10. Scale bar: 100 μm. **(b)** Quantitative distribution of cell morphology (elongated, fried-egg, and rounded/immature) under each protocol, expressed as the percent of cells. Data are presented as mean ± standard deviation (SD). **(c)** Representative Giemsa-stained images of cells differentiated under each protocol (P1, M-CSF-based medium; P2, nutrient-enriched cytokine-free medium; P3, commercial human M1-polarizing medium; and P4, commercial human M2-polarizing medium). Scale bar: 100 μm.

Distinct differences in cell morphology, adhesion, and proliferation were observed across the four culture protocols (Figure 1a). Initially, cells cultured under all conditions appeared small and round. By day 3, cells obtained using P2 exhibited the most rapid differentiation, displaying elongated or “fried egg”-like shapes, followed by P1. In contrast, cells from P3 and P4 remained largely round and loosely attached, indicating delayed differentiation. By day 10, quantitative analysis confirmed that P2 showed a higher proportion of elongated cells compared to other conditions, indicating relatively enhanced morphological differentiation under the present experimental conditions (Figure 1b). P2 yielded the highest proportion of elongated macrophages (41.67 ± 10.41%), surpassing P3 and P4 (*p* = 0.006), which were dominated by rounded, immature cells (over 70%). Although the “fried-egg” morphology was prevalent under both P2 (50.00%) and P1 (48.33%), it was minimal under P3 and P4 (*p* = 0.0001). Giemsa staining corroborated these findings (Figure 1c), indicating that all groups exhibited key morphological features of macrophages, including an increased cytoplasm-to-nucleus ratio, the presence of pseudopodia, and the development of vacuolar structures associated with phagocytic activity. However, the consistency and degree of maturation varied across protocols.

### 3.2 Transcriptional profiling of macrophage markers

**Figure 2:**
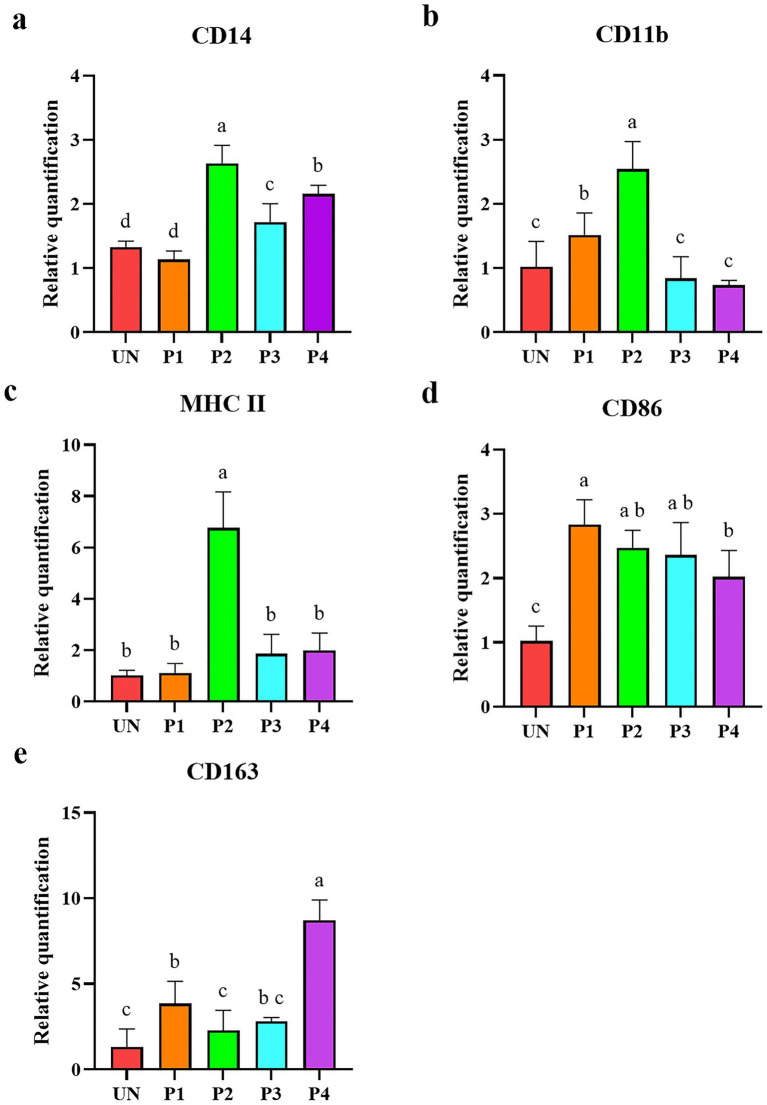
Gene expression analysis of macrophage surface markers under different differentiation protocols. Relative mRNA expression of macrophage surface and differentiation markers was evaluated using RT-qPCR in undifferentiated cells (UN) and cells differentiated using four protocols (P1, M-CSF-based medium; P2, nutrient-enriched cytokine-free medium; P3, commercial human M1-polarizing medium; and P4, commercial human M2-polarizing medium). Expression levels were normalized to those of *ACTB*. In the bar graphs, red indicates UN, orange indicates P1, green indicates P2, sky blue indicates P3, and purple indicates P4. Panels show the relative expression of **(a)** *CD14*, **(b)** *CD11b*, **(c)** *MHC II*, **(d)** *CD86*, and **(e)** *CD163*. Bars represent the mean ± SD of values from five independent experiments, and different letters **(a–d)** above bars indicate statistically significant differences at *p* < 0.05, as determined using a one-way ANOVA followed by Tukey’s post-hoc test. RT-qPCR, quantitative reverse transcription polymerase chain reaction; SD, standard deviation; ANOVA, analysis of variance.

RT-qPCR was conducted to evaluate the transcriptional profiles of macrophage-related genes in undifferentiated PBMCs (UN), which served as the control; macrophages were allowed to differentiate under four distinct culture protocols (Figure 2). Regarding monocyte/macrophage lineage markers, P2 induced the most robust mRNA upregulation of both *CD14* and *CD11b* (Figures 2a,b). Although these markers were upregulated under other protocols, *CD14* expression under P1 and *CD11b* expression under P3 and P4 were similar to the UN control. Regarding antigen presentation–related genes, *MHC II* transcripts were strongly increased exclusively under P2, whereas transcripts under P1, P3, and P4 displayed expression levels comparable to those of the UN control (Figure 2c).

An analysis of polarization-associated markers indicated that the M1-related gene, *CD86*, was upregulated in all differentiated groups compared with that in PBMCs (Figure 2d). Although P1 yielded the highest numeric value for the increase in *CD86* expression, it was not statistically different from that for P2 and P3, whereas P4 showed a relatively lower increase. In contrast, the M2-associated gene, *CD163*, was most prominently induced under P4 (Figure 2e). *CD163* showed moderate expression under P1, and its expression under P2 was not statistically different from that in the UN control. Collectively, these results indicate that P2 could effectively promote macrophage differentiation as characterized by robust gene expression of general markers, including *CD14*, *CD11b*, and *MHC II*, whereas P1 and P4 favored M1- and M2-polarized profiles, respectively.

### 3.3 Surface protein expression of macrophage markers

**Figure 3:**
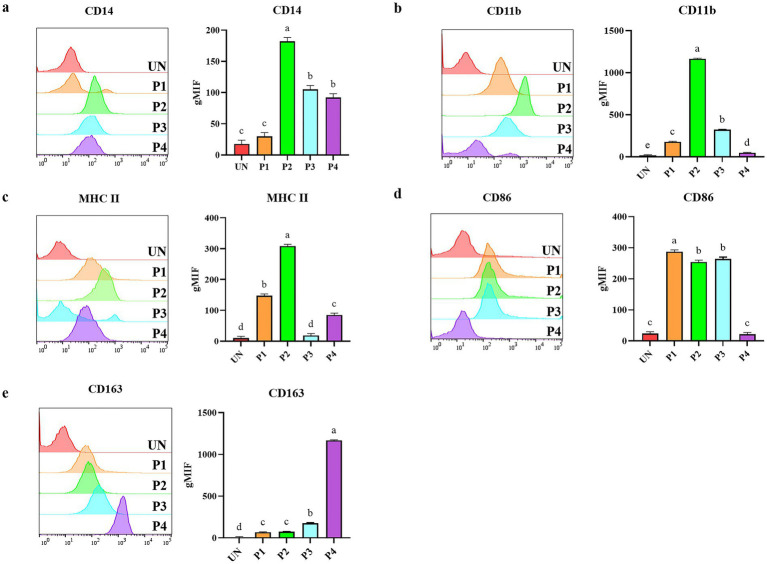
Flow cytometric analysis of macrophage surface marker expression under different differentiation protocols. Flow cytometry was conducted to assess the surface expression of macrophage markers in undifferentiated cells (UN) and cells differentiated using four protocols (P1, M-CSF-based medium; P2, nutrient-enriched cytokine-free medium; P3, commercial human M1-polarizing medium; and P4, commercial human M2-polarizing medium). Each marker is shown as a half-overlay histogram (left) and the corresponding geometric mean fluorescence intensity (gMFI) bar graph (right). In both histograms and bar graphs, red indicates UN, orange indicates P1, green indicates P2, sky blue indicates P3, and purple indicates P4. Panels show the expression of **(a)** CD14, **(b)** CD11b, **(c)** MHC II, **(d)** CD86, and **(e)** CD163. The gMFI data are presented as the mean ± SD of values from three independent experiments. Statistical differences among groups were evaluated using a one-way ANOVA followed by Tukey’s post-hoc test; different letters **(a–e)** above the bars indicate significant differences at *p* < 0.05. gMFI, geometric mean fluorescence intensity; SD, standard deviation; ANOVA, analysis of variance.

To phenotypically characterize the *in vitro* PBMC-MDMs, the expression of macrophage activation-related surface markers was assessed on day 10 (Figure 3). Of the differentiation protocols, P2 induced the most robust expression of the monocyte/macrophage lineage markers, CD14 and CD11b, as well as the antigen-presenting molecule, MHC II, with substantially higher gMFI values than other groups (Figures 3a–c). Whereas P1 and P3 generally resulted in upregulated levels of these markers compared to those in the UN control, CD14 expression in the P1 group and MHC II expression in the P3 group did not differ substantially from that in the UN control. Regarding polarization markers, the expression of the M1-associated costimulatory molecule, CD86, peaked in the P1 group, followed by that in the P2 and P3 groups, whereas P4 failed to induce marked upregulation compared to UN (Figure 3d). In contrast, the M2 marker, CD163, was predominantly expressed in the P4 group, indicating the specific induction of an M2-like phenotype (Figure 3e). Collectively, these results demonstrate that P2 generated the most mature and immunologically competent phenotype, characterized by a high antigen-presenting capacity. In contrast, P1 and P3 cells showed intermediate differentiation with variable marker profiles, whereas P4 cells skewed towards an M2-polarized profile with limited maturation. The percentage of marker-positive cells for each surface marker is summarized in Supplementary Table 1.

### 3.4 Functional assessment of phagocytic capacity

**Figure 4:**
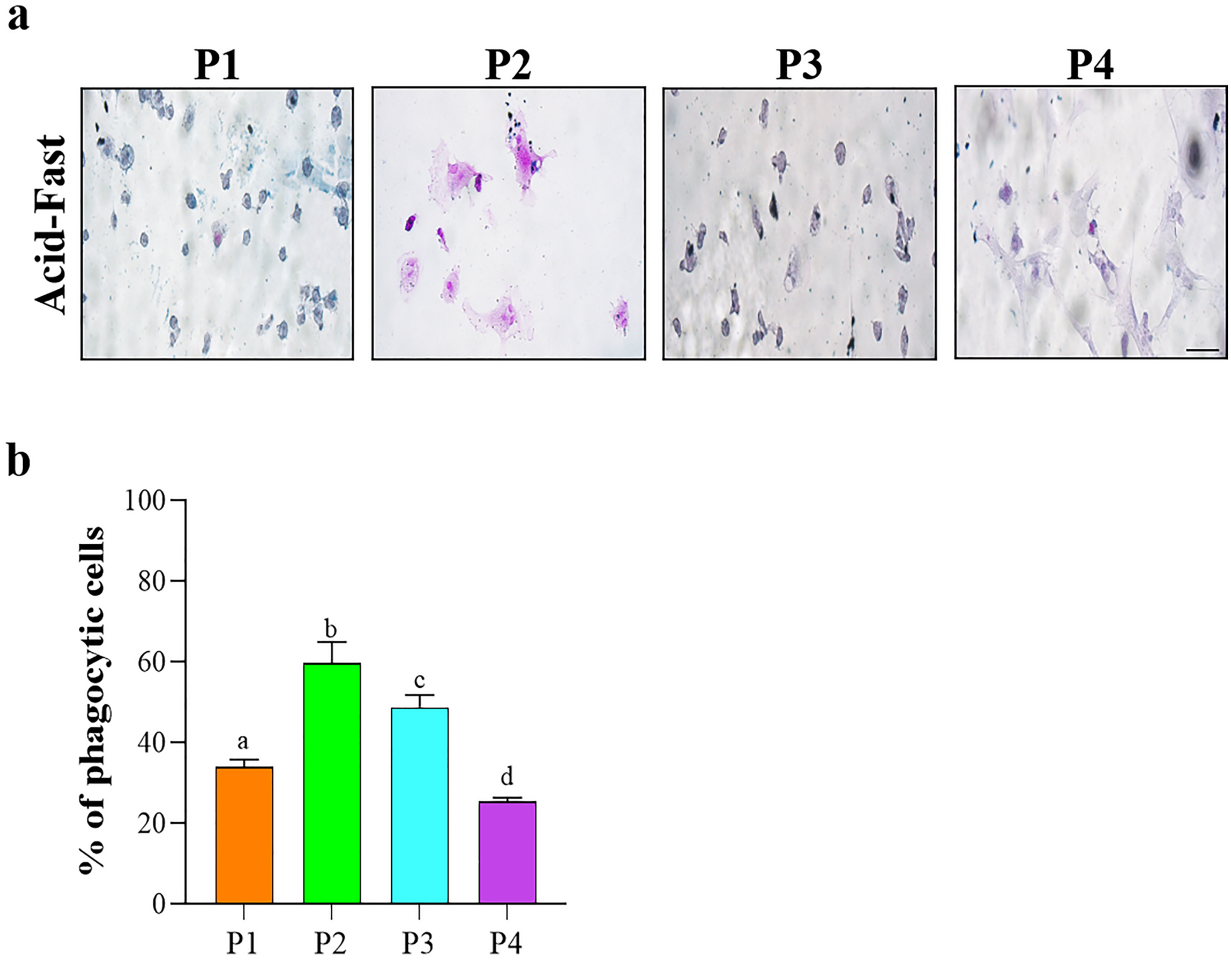
Evaluation of macrophage infection and phagocytic activity under different conditions. **(a)** Representative images of macrophages differentiated using each protocol (P1, M-CSF-based medium; P2, nutrient-enriched cytokine-free medium; P3, commercial human M1-polarizing medium; and P4, commercial human M2-polarizing medium) and infected with MAP, visualized using Ziehl–Neelsen staining (acid-fast bacteria in red against a blue background). **(b)** Phagocytic activity of macrophages differentiated using each protocol (P1, M-CSF-based medium; P2, nutrient-enriched cytokine-free medium; P3, commercial human M1-polarizing medium; and P4, commercial human M2-polarizing medium), expressed as the percentage of phagocytic cells. Data are presented as the mean ± SD of values from three independent experiments. Different letters (a–d) indicate statistically significant differences at *p* < 0.05, determined using a one-way ANOVA followed by Tukey’s post-hoc test. MAP, *Mycobacterium avium* subsp. *Paratuberculosis*; SD, standard deviation; ANOVA, analysis of variance.

To validate the phagocytic capacity of the differentiated macrophages, cells were infected with MAP and visualized using Ziehl–Neelsen staining. Acid-fast bacilli were detected intracellularly in all experimental groups post-infection, confirming the successful uptake of MAP (Figure 4a). A quantitative analysis of phagocytic activity indicated distinct differences among the groups (Figure 4b). P2 yielded (59%) the highest phagocytic rate, sequentially followed by P3 (48%), P1 (34%), and P4 (25%). P2 resulted in the highest phagocytic rate, demonstrating superior functional capacity compared to other conditions. These data confirm that the nutrient-enriched conditions of P2 not only promoted phenotypic maturation but also distinctively enhanced the functional ability of PBMC-MDMs to internalize pathogens.

## 4 Discussion

In this study, four culture conditions were compared for their ability to support the differentiation of bovine PBMCs into macrophages with distinct morphological, phenotypic, and phagocytic characteristics. The nutrient-enriched P2 medium preferentially generated macrophages characterized by pronounced morphological differentiation, robust lineage marker expression, and relatively high phagocytic activity against MAP under the present experimental conditions.

The comparative analysis demonstrated that the intrinsic nutrient composition of the culture medium was the primary driver of differentiation efficacy. Of the tested groups, the P2 condition promoted the most rapid morphological changes while consistently conferring the cells with the characteristic spindle-shaped and fried egg-like features typical of macrophages. Previous research on human monocytes has established that specific growth factors drive distinct morphologies, wherein M-CSF typically induces a spindle shape, and GM-CSF promotes a fried egg appearance (28). Moreover, monocyte-derived macrophages are known to exhibit heterogeneous morphological and functional phenotypes depending on differentiation conditions and cytokine exposure (15). Bovine macrophages in the P2 group exhibited a mixed population displaying both of these characteristics even without exogenous M-CSF and GM-CSF. This autonomous phenotypic development is likely supported by the enriched nutrient profile of the P2 medium, which included amino acids, pyruvate, and 2-mercaptoethanol. Recent studies have suggested that macrophage differentiation and polarization are closely linked to metabolic reprogramming and nutrient availability within the microenvironment (16). According to recent metabolic studies, these components are essential for supporting biosynthetic metabolism and maintaining intracellular glutathione homeostasis (29, 30), thereby contributing to monocyte-to-macrophage differentiation and the expression of macrophage-associated markers.

Complementing these morphological observations, flow cytometric analysis indicated that P2 cells did not fit a simple binary polarization paradigm but instead differentiated into a heterogeneous population co-expressing M1 (MHC II, CD86) and M2 (CD163) markers. This observation aligns with previous studies highlighting species-specific differentiation pathways and the substantial phenotypic plasticity of bovine monocyte-derived macrophages (10, 11, 31). This observation aligns with a previous study highlighting species-specific differentiation pathways, which reported that bovine monocytes naturally acquire a mixed M1/M2 phenotype even under conditions that drive human monocytes toward a strict pro-inflammatory M1 state, such as exposure to neutrophil degranulation products. Despite this heterogeneity, the P2 condition induced the most robust upregulation of surface markers critical for pathogen interaction in comparison to other protocols. Specifically, P2 cells exhibited the highest expression of CD14, CD11b, and MHC II. CD14 mediates the internalization of non-opsonized mycobacteria and Toll-like receptor signaling, whereas CD11b (CR3) facilitates phagocytic uptake by directly recognizing cell wall glycolipids, such as LAM and PIM (32). This receptor profile may contribute to the relatively enhanced phagocytic activity observed in P2 cells under the present experimental conditions, as evidenced by their higher uptake of MAP compared with the other differentiation protocols. Such functional diversity among bovine macrophage populations has also been described previously, where distinct activation states were associated with differential inflammatory and antimicrobial responses (9). Furthermore, the concurrent expression of MHC II and the co-stimulatory molecule CD86 suggests that P2-derived macrophages may retain antigen presentation-associated characteristics potentially relevant to downstream adaptive immune responses. Collectively, these findings support the utility of the direct plastic adherence approach without prior CD14 + enrichment, consistent with previously established bovine macrophage protocols (13, 33). Although CD14 + magnetic sorting is commonly used to increase monocyte purity (34, 35), we intentionally employed the direct adherence method to preserve phenotypic heterogeneity and avoid potential artificial activation induced by antibody ligation (36), while also retaining biologically relevant CD14-low monocyte subsets (10). This differentiation model is particularly relevant in the context of bovine paratuberculosis caused by MAP. MAP is a facultative intracellular pathogen that survives and persists primarily within macrophages, where it modulates host immune signaling and evades bactericidal responses (20, 21). Therefore, the biological characteristics of *in vitro*-generated bovine macrophages can substantially influence the interpretation of MAP–host interaction studies. In particular, differences in macrophage differentiation status, receptor expression, and polarization phenotype may affect bacterial uptake, intracellular survival, cytokine responses, antigen presentation, and downstream immune activation (12–14). Furthermore, macrophage differentiation pathways and cytokine responsiveness differ substantially between species (10, 37, 38), limiting the direct applicability of human- or murine-derived protocols to bovine systems. Establishing a reproducible bovine PBMC-derived macrophage model may therefore provide a more physiologically relevant platform for investigating the early immunopathological mechanisms of MAP infection, host–pathogen interactions, and biomarker discovery in cattle (13, 19, 33).

In contrast to the differentiation pattern observed under the nutrient-enriched P2 condition, protocols relying on exogenous cytokines or human-optimized media generated distinct morphological and phenotypic characteristics. Exogenous colony-stimulating factors, particularly M-CSF and GM-CSF, are standard for driving macrophage differentiation in human and murine models (39). In the P1 protocol, recombinant M-CSF was used, anticipating it would induce a homeostatic phenotype similar to that of tissue-resident macrophages targeted by MAP rather than GM-CSF (40). However, P1 cells displayed a CD14-/CD86+/MHC II + profile, distinct from the classical CD14 + macrophages observed in P2 cells. This CD14^−^/CD86+/MHC II^+^ profile in P1 cells closely resembles the phenotype of bovine afferent lymph macrophages described by Guzman et al. (41). The loss of CD14 expression suggests that exogenous M-CSF may bias bovine monocyte differentiation towards an antigen-presenting phenotype with reduced scavenging capacity, rather than maintaining the classical CD14 + macrophage identity.

The commercial media used in P3 and P4, which are optimized for human cells, resulted in delayed morphological maturation and considerable cellular heterogeneity. Specifically, P3 failed to substantially upregulate MHC II, a hallmark of M1 polarization, despite being explicitly formulated for this purpose. Similarly, although P4 induced high levels of *CD163* transcripts and surface protein, the cells remained morphologically round and exhibited the lowest phagocytic activity. These findings suggest that the commercial M2 media might induce partial marker expression without triggering the cytoskeletal rearrangements and functional machinery required for full maturation. This disconnect highlights the risk of relying solely on human-optimized reagents for bovine research. These suboptimal outcomes suggest that cross-species cytokine reactivity is limited by structural homology (38). Specifically, human GM-CSF exhibits reduced specific activity on bovine progenitor cells compared with that on human cells (37). Considering the species-specific differences in cytokine structure and receptor interactions, we interpret that the heterogeneous phenotypes observed under the P3 and P4 conditions likely represent bovine-specific responses to heterologous cytokine stimulation rather than incomplete differentiation. Accordingly, the inefficiency of human-specific formulations in our study reinforces the necessity of developing tailored culture conditions that account for species-specific nutritional and signaling requirements.

RT-qPCR analysis generally corroborated the phenotypic profiles established using flow cytometry, confirming the robust identity of P2 macrophages (high CD14, CD11b, MHC II transcripts) and the M2-like polarization of P4 macrophages (high CD163 transcripts). However, discrepancies were noted between the transcript and protein levels of specific markers. For instance, whereas P1 and P4 groups displayed clear surface protein expression of MHC II (+), the mRNA levels were negligible. These inconsistencies can be attributed to post-transcriptional regulation or the kinetics of protein stability (42). Conversely, in the P1 group, *CD163* mRNA was upregulated (+), yet surface protein expression remained low. The low surface detection of CD163 despite high transcriptional activity may result from ectodomain shedding, a process wherein surface receptors are cleaved into soluble forms (sCD163) upon strong cellular activation or inflammation (43). Given these dynamic regulatory mechanisms, surface protein expression was prioritized as the definitive indicator of functional cell status. The assessment of macrophage differentiation and phenotypic maturity was based primarily on the flow cytometric profiles, which directly reflect the availability of functional receptors on the cell surface.

Despite the clear advantages of the optimized protocol, this study has several limitations. First, the precise molecular mechanisms underlying differentiation under the nutrient-enriched P2 condition remain undefined. Second, as different media formulations may exhibit distinct differentiation kinetics in bovine cells, the standardized 10-day culture period may not be optimal for all protocols. Therefore, additional time-course studies would be valuable to determine the optimal differentiation period for each protocol under bovine-specific conditions. In addition, the ImageJ-based quantification of cell morphology and phagocytosis, as well as the relatively wide age range and unbalanced sex distribution of the animals used in this study, may have contributed to observer-dependent interpretation and biological variability, respectively. Regarding flow cytometric analysis, the absence of a viability dye represents a methodological limitation. Although the impact of dead cells was minimized through thorough physical washing and stringent scatter-based gating, incorporating a viability dye remains the gold standard to entirely prevent non-specific binding. Finally, the functional evaluation in this study focused primarily on phagocytic uptake. Therefore, further functional assays assessing cytokine secretion, antigen presentation capacity, reactive oxygen species/nitric oxide production, and microbial killing would be necessary to comprehensively define the functional status of macrophages generated under each culture condition.

Nevertheless, this study presents a substantial methodological advance by establishing a highly accessible and cytokine-free bovine macrophage differentiation model. Unlike bone marrow-based systems that require invasive procedures or animal sacrifice, the current protocol uses PBMCs obtained through simple blood collection and enables functional macrophage differentiation solely through nutrient enrichment. This approach provides a practical and cost-effective alternative to recombinant cytokine supplementation or commercially available human-oriented media. Collectively, this study establishes a scalable framework for bovine macrophage research and provides a useful platform for investigating host–pathogen interactions, particularly in the context of bovine paratuberculosis and MAP-associated immunopathogenesis.

## Data Availability

The original contributions presented in the study are included in the article/Supplementary material, further inquiries can be directed to the corresponding author.
