# Supplementary material for: Comparison of four culture protocols for differentiating bovine peripheral blood mononuclear cells into macrophages
Source: Front Vet Sci. 2026 Jun 23;13:1851348. doi: 10.3389/fvets.2026.1851348 (PMC13337438; doi:10.3389/fvets.2026.1851348)
Supplement: Supplementary file 1 [file Table_1.DOCX]

**Supplementary Materials**

**Supplementary Table 1.** Percentage of marker-positive cells among peripheral blood mononuclear cells-derived macrophages differentiated under four culture conditions

| Marker-positive cells (%) | UN | P1 | P2 | P3 | P4 |
| --- | --- | --- | --- | --- | --- |
| *CD14* | 4.23 ± 4.42ᶜ | 17.23 ± 4.43ᶜ | 89.52 ± 4.43ᵃ | 57.64 ± 4.43ᵇ | 51.52 ± 4.43ᵇ |
| *CD11b* | 4.43 ± 4.42ᵈ | 70.22 ± 0.47ᵇ | 88.48 ± 5.56ᵃ | 74.23 ± 1.92ᵇ | 24.06 ± 4.43ᶜ |
| *MHC II* | 6.26 ± 4.42ᵈ | 47.09 ± 5.06ᵇ | 79.21 ± 7.83ᵃ | 18.11 ± 4.46ᵈ | 30.93 ± 9.25ᶜ |
| *CD86* | 16.91 ± 4.42ᶜ | 91.39 ± 7.85ᵃ | 88.96 ± 7.85ᵇ | 90.55 ± 7.85ᵇ | 4.88 ± 4.43ᶜ |
| *CD163* | 4.96 ± 4.43ᵈ | 38.72 ± 5.86ᶜ | 51.97 ± 5.86ᵇ | 52.09 ± 5.86ᵇ | 67.75 ± 2.51ᵃ |

P1, M-CSF-based medium; P2, nutrient-enriched cytokine-free medium; P3, commercial human M1-polarizing medium; and P4, commercial human M2-polarizing medium. Abbreviations: UN, undifferentiated PBMCs; P1, protocol 1; P2, protocol 2; P3, protocol 3; P4, protocol 4; SD, standard deviation; ANOVA, analysis of variance.
